# A Proposed Clinical Validation Pathway for the Artificial Intelligence-Driven Integrated Risk Assessment of Cardiovascular Disease (AIRA-CVD): A Translational Framework Integrating Inflammatory Biomarkers, Histopathology, and Machine Learning

**DOI:** 10.7759/cureus.111315

**Published:** 2026-06-22

**Authors:** CFC Ogbuefi

**Affiliations:** 1 Department of Family Medicine, Federal University Teaching Hospital, Owerri, NGA

**Keywords:** artificial intelligence, cardiovascular disease, health informatics, histopathology, inflammatory biomarkers, machine learning, precision medicine, predictive analytics, risk assessment

## Abstract

Cardiovascular disease (CVD) remains a leading cause of morbidity, mortality, and healthcare expenditure despite advances in prevention and treatment. The Artificial Intelligence-Driven Integrated Risk Assessment of Cardiovascular Disease (AIRA-CVD) framework was proposed as a multimodal architecture integrating clinical data, inflammatory biomarkers, imaging findings, and histopathological evidence of vascular remodeling to support precision cardiovascular risk assessment. This technical report presents a structured clinical validation pathway designed to facilitate future implementation and evaluation of the framework. The proposed pathway consists of four sequential phases: retrospective electronic health record analysis, inflammatory biomarker integration, histopathological validation, and prospective clinical implementation. The framework further incorporates a hybrid machine learning architecture, explainable artificial intelligence methodologies, human-in-the-loop clinical oversight, and algorithmic fairness considerations to support transparency, interpretability, and responsible deployment. Potential applications include enhanced cardiovascular risk stratification, earlier identification of high-risk individuals, support for preventive interventions, and integration within clinical decision-support and population health management systems. By providing a translational roadmap for validation and implementation, this report seeks to bridge the gap between computational innovation and patient-centered cardiovascular care while advancing the development of biologically informed artificial intelligence systems in cardiovascular medicine.

## Introduction

Cardiovascular disease (CVD) remains the leading cause of death in the United States and represents one of the most significant challenges facing modern healthcare systems [1]. Despite substantial advances in pharmacologic therapies, diagnostic imaging, and preventive medicine, CVD continues to account for hundreds of thousands of deaths annually while generating enormous healthcare expenditures and productivity losses [2,3]. The burden of CVD is expected to increase further as the population ages and the prevalence of obesity, diabetes mellitus, hypertension, and metabolic syndrome continues to rise [3].

Accurate identification of high-risk individuals remains central to cardiovascular prevention. Traditional risk assessment tools such as the Framingham Risk Score and the pooled cohort equations have played important roles in guiding preventive interventions [4]. However, these models rely primarily on conventional clinical variables and population-derived risk estimates [4]. As a result, they may inadequately capture biological heterogeneity, inflammatory burden, and early pathophysiological changes that precede clinical cardiovascular events.

Increasing evidence suggests that chronic inflammation plays a critical role throughout the progression of CVD [5]. Inflammatory mediators contribute to endothelial dysfunction, atherosclerotic plaque formation, vascular remodeling, and plaque instability [5]. Biomarkers such as high-sensitivity C-reactive protein (hs-CRP) and interleukin-6 (IL-6) have demonstrated significant associations with future cardiovascular events independent of traditional risk factors [6]. These observations have led investigators to explore inflammation-based approaches to cardiovascular risk prediction and prevention [7,8].

Simultaneously, advances in artificial intelligence (AI) and machine learning have transformed the ability of healthcare systems to analyze large and complex datasets [9]. Modern AI systems can integrate information from electronic health records (EHRs), diagnostic imaging, laboratory testing, wearable devices, and genomic platforms to generate individualized risk predictions [10]. Within cardiovascular medicine, machine learning algorithms have demonstrated promising capabilities in arrhythmia detection, heart failure prediction, imaging interpretation, and risk stratification [11].

In a previously published technical framework, the Artificial Intelligence-Driven Integrated Risk Assessment of Cardiovascular Disease (AIRA-CVD) architecture was proposed as a multimodal platform integrating clinical informatics, inflammatory biomarkers, imaging data, and histopathological evidence of vascular remodeling [12]. Subsequent publications further explored ethical governance considerations for cardiovascular AI and the broader role of lifestyle and population health determinants in CVD prevention [13,14]. While the framework established the theoretical and technical foundations of the model, successful implementation requires a structured translational strategy capable of validating predictive performance while maintaining clinical relevance, interpretability, and equity.

The purpose of this technical report is to propose a comprehensive clinical validation pathway for the AIRA-CVD framework. Specifically, this report describes a phased translational strategy designed to evaluate predictive performance, establish biological validity, and support eventual implementation within clinical and public health settings.

The AIRA-CVD framework was previously introduced as a conceptual multimodal architecture integrating clinical variables, inflammatory biomarkers, histopathological evidence, and AI to improve cardiovascular risk assessment [12]. The present report differs from the original publication by proposing a structured translational pathway for future clinical validation rather than reporting completed validation results. Specifically, this technical report outlines the sequential processes required for retrospective model development, biomarker integration, biological verification, external validation, and prospective clinical implementation. By clearly distinguishing conceptual framework development from empirical validation, this article provides researchers and clinicians with a practical roadmap for advancing biologically informed AI toward safe and clinically applicable cardiovascular risk prediction.

## Technical report

Study design and translational validation framework

The proposed AIRA-CVD validation framework is designed to facilitate the translation of the previously published conceptual architecture into a clinically applicable cardiovascular risk assessment platform [12]. The framework consists of four sequential and interdependent phases that collectively evaluate predictive performance, biological validity, clinical utility, and implementation feasibility. Each phase builds upon the preceding stage, creating a structured pathway from retrospective model development to prospective clinical application.

Phase I focuses on retrospective model development using de-identified EHR datasets. Clinical variables, including demographic characteristics, cardiovascular risk factors, laboratory measurements, medication histories, and imaging findings, are incorporated into predictive modeling workflows. Machine learning algorithms are applied to identify patterns associated with major adverse cardiovascular events and establish baseline predictive performance.

Phase II introduces inflammatory biomarker integration. Biomarkers, including hs-CRP and IL-6, are incorporated into predictive models to provide insight into underlying vascular inflammation and disease activity [6-8]. This phase seeks to enhance biological sensitivity and improve identification of individuals with subclinical CVD who may not be recognized through conventional risk assessment approaches alone.

Phase III focuses on histopathological validation, which is intended as a biological verification strategy rather than a routine diagnostic procedure. Tissue analyses would be performed only when vascular specimens become available through clinically indicated procedures, including carotid endarterectomy, coronary atherectomy, or other vascular interventions, thereby avoiding unnecessary invasive testing while providing biological correlation between model predictions and vascular pathology. Histopathological features such as intimal thickening, vascular fibrosis, inflammatory cell infiltration, endothelial injury, and atherosclerotic plaque development may serve as biological reference standards for evaluating predictive outputs [15,16]. By linking computational predictions to measurable tissue-level pathology, this phase seeks to strengthen interpretability and biological plausibility.

Phase IV involves prospective clinical validation and implementation. During this phase, the framework would be evaluated within real-world healthcare environments and compared with existing cardiovascular risk assessment approaches. The objective is to determine whether integration of inflammatory biomarkers, machine learning analytics, and biological validation strategies can support more precise cardiovascular risk stratification while maintaining appropriate clinical oversight (Table 1).

**Table 1:** Proposed four-phase clinical validation pathway for AIRA-CVD. The proposed validation framework consists of four sequential phases designed to evaluate predictive performance, biological plausibility, and clinical utility. Phase I focuses on retrospective machine learning model development using electronic health record data. Phase II incorporates inflammatory biomarkers associated with cardiovascular risk. Phase III correlates predictive outputs with histopathological evidence of vascular remodeling. Phase IV evaluates clinical implementation and real-world applicability. AIRA-CVD = Artificial Intelligence-Driven Integrated Risk Assessment of Cardiovascular Disease; hs-CRP = high-sensitivity C-reactive protein; IL-6 = interleukin-6.

Phase	Objective	Primary data source
Phase I	Retrospective model development and performance evaluation	Electronic health records, laboratory and imaging data
Phase II	Integration of inflammatory biomarkers	hs-CRP, IL-6, and related inflammatory markers [6-8]
Phase III	Biological validation	Histopathological vascular remodeling data [15,16]
Phase IV	Prospective clinical implementation	Real-world cardiovascular patient populations

The proposed validation pathway is intended for adult populations undergoing cardiovascular risk assessment without established atherosclerotic CVD at baseline. Future validation studies would evaluate major adverse cardiovascular events, including myocardial infarction, ischemic stroke, cardiovascular death, and coronary revascularization over predefined five-year and 10-year prediction horizons. Model performance would be compared against established cardiovascular risk prediction tools, including the Framingham Risk Score and the pooled cohort equations, using discrimination, calibration, clinical utility, and external validation across independent healthcare systems. Missing clinical variables would be addressed using established multiple-imputation techniques where appropriate, while decision-curve analysis and calibration assessment would evaluate potential clinical usefulness before prospective implementation.

Machine Learning Architecture and Computational Verification Protocols

To support future computational validation of the proposed AIRA-CVD framework, phase I validation would include independent performance stress-testing across its discrete algorithmic components. Tabular pipelines utilizing ensemble gradient boosting methods will be audited using stratified train-test splits and k-fold cross-validation to guarantee baseline prediction stability on highly dimensional clinical datasets. Concurrently, the convolutional neural network (CNN) modules responsible for imaging interpretation will undergo automated testing against diverse multi-institutional imaging repositories to verify the consistency of structural feature extraction from computed tomography (CT) angiograms and echocardiograms. Performance calibration across these distinct data streams will be quantified using area under the receiver operating characteristic curve (AUC-ROC) benchmarks, precision-recall curves, and Brier scores. Furthermore, the local explainability layer will be audited by tracking the consistency of SHapley Additive exPlanations (SHAP) feature attributions across varying patient cohorts to ensure that the computational rationale remains transparent and easily interpretable by clinical end-users.

Operational Framework for Human-in-the-Loop Oversight

The validation pathway treats human-in-the-loop interaction not as an external regulatory requirement but as a dynamic feedback loop that directly impacts model stability. Phase IV implementation protocols establish structured evaluation interfaces where clinicians interact directly with the framework’s decision-support outputs. During this phase, the agreement rate between algorithmic classifications and independent physician assessments will be measured to define precise boundaries for automated risk stratification. Explainable AI visualizations, including dynamic feature-contribution plots, will be tested to ensure they effectively communicate complex risk vectors in high-acuity environments without inducing cognitive overload or clinician alert-fatigue. Additionally, the pathway incorporates a longitudinal feedback reporting system, allowing provider adjustments and discrepancies to be securely cataloged to inform subsequent iterations of model fine-tuning and performance auditing.

Algorithmic Fairness and Health Equity Verification Metrics

**Figure 1:**
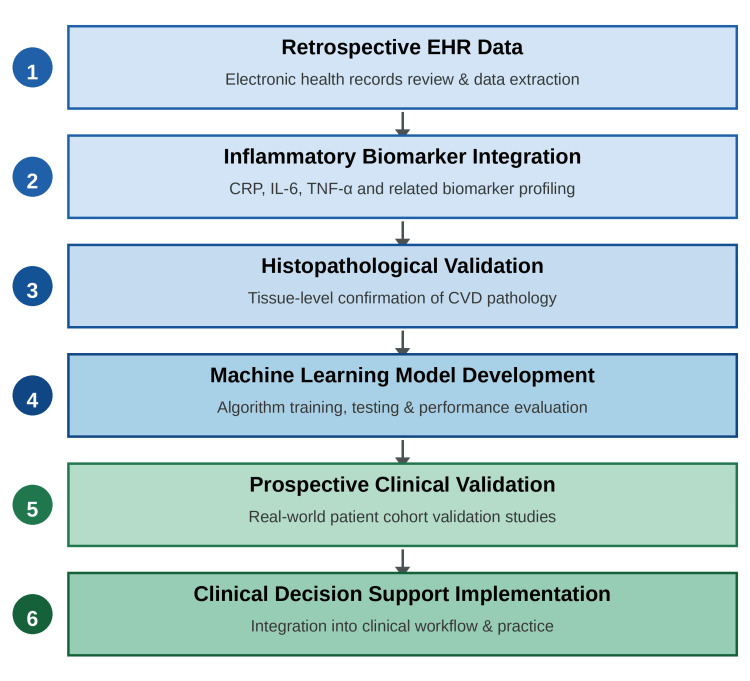
Proposed clinical validation pathway for AIRA-CVD. The proposed validation framework progresses from retrospective electronic health record analysis and machine learning model development to inflammatory biomarker integration, histopathological validation, and prospective clinical implementation. Phase III tissue analysis is designed as an opportunistic verification layer utilizing specimens obtained strictly during clinically indicated vascular interventions, avoiding unnecessary invasive testing. The framework is designed to support biological interpretability, predictive accuracy, and responsible deployment of artificial intelligence within cardiovascular risk assessment. Image Credits: CFC Ogbuefi. Created manually using Python Matplotlib (Python Software Foundation, Wilmington, DE, USA). AIRA-CVD: Artificial Intelligence-Driven Integrated Risk Assessment of Cardiovascular Disease; EHR: electronic health records; CRP: C-reactive protein; IL-6: interleukin-6; TNF-α: tumor necrosis factor alpha; CVD: cardiovascular disease.

Given that historical healthcare infrastructure contains deep-seated systemic inequities, the validation architecture prioritizes the proactive identification and mitigation of algorithmic bias [17]. The equity auditing framework utilizes strict statistical measures, specifically demographic parity difference and equal opportunity difference, to evaluate model behavior across distinct demographic, socioeconomic, and geographic sub-populations [17,18]. As illustrated in the validation workflow schema (Figure 1), data validation splits will undergo deep slicing rather than relying on population-level averages to isolate predictive performance within historically underserved and marginalized communities [19]. If unexpected calibration drift or unequal error rates are detected across racial or sex boundaries, automated data reweighting and specialized fairness-aware loss functions will be triggered within the retraining loop to equalize sensitivity. By enforcing this continuous equity surveillance protocol, the framework establishes a reproducible standard for validating that predictive analytics actively reduce, rather than amplify, existing health disparities in preventive cardiology.

Proposed validation pathway and anticipated outcomes

As a conceptual translational framework, this report does not present empirical clinical validation data or proof-of-concept simulations. Instead, it outlines the anticipated validation pathway, proposed implementation strategy, and expected clinical applications that future prospective studies should evaluate. The objective is to establish a reproducible roadmap for systematic validation rather than to report completed model performance.

Anticipated Predictive Advantages

The AIRA-CVD framework is designed to improve cardiovascular risk assessment through the integration of clinical variables, inflammatory biomarkers, histopathological validation, and machine learning analytics [12]. Unlike conventional risk calculators that rely primarily on demographic and clinical factors, the proposed framework incorporates biological indicators of vascular inflammation and disease progression [5,6]. This multimodal approach may improve identification of individuals with subclinical CVD, support earlier intervention, and enhance individualized risk stratification. The incorporation of histopathological validation further distinguishes the framework by linking computational predictions to measurable biological evidence of vascular injury [15,16].

Hypothetical Clinical Application

A potential application of the framework may involve a patient classified as having intermediate cardiovascular risk according to traditional risk assessment models [4]. Although conventional risk factors alone may not warrant intensive intervention, persistently elevated hs-CRP and IL-6 concentrations could indicate ongoing vascular inflammation [6,7]. By integrating biomarker information with longitudinal clinical data and imaging findings, the AIRA-CVD framework may identify patterns associated with increased cardiovascular risk that might otherwise remain undetected. Such information could assist clinicians in considering additional diagnostic evaluation or preventive strategies while maintaining appropriate clinical oversight.

Potential Public Health Applications

If successfully validated, the AIRA-CVD framework may contribute to broader CVD prevention efforts by supporting earlier identification of high-risk individuals and improving allocation of preventive resources. Enhanced risk prediction could facilitate targeted interventions among populations at elevated risk for myocardial infarction, stroke, and other adverse cardiovascular outcomes [3]. The framework may also support ongoing public health initiatives aimed at reducing cardiovascular morbidity, mortality, and healthcare expenditures through more effective prevention and risk-stratification strategies [2,20]. The framework may complement existing national cardiovascular prevention initiatives, including programs focused on improving early detection and risk-factor management among high-risk populations [20].

Potential Integration Within Healthcare Systems

The proposed framework is designed to function within existing healthcare infrastructures and may be incorporated into EHR-based decision-support systems, preventive cardiology programs, academic medical centers, and population health management initiatives. Integration within these environments could facilitate routine cardiovascular risk assessment while supporting clinician-guided interpretation of predictive outputs. As healthcare data ecosystems continue to evolve, the framework may also accommodate emerging data sources, including wearable health technologies, remote patient monitoring platforms, and advanced diagnostic tools.

## Discussion

The AIRA-CVD framework was previously introduced as a conceptual architecture integrating clinical cardiovascular data, inflammatory biomarkers, imaging findings, and histopathological evidence of vascular remodeling to support precision cardiovascular risk assessment [12]. The present technical report extends that work by proposing a structured clinical validation pathway designed to facilitate future translation from conceptual development to clinical implementation.

Although hs-CRP and IL-6 remain among the most extensively studied inflammatory biomarkers, recent evidence supports the expanding role of additional biomarkers in cardiovascular risk assessment. These include high-sensitivity cardiac troponins, N-terminal pro-B-type natriuretic peptide, growth differentiation factor-15, galectin-3, soluble suppression of tumorigenicity-2, myeloperoxidase, apolipoprotein B, and lipoprotein(a), all of which have demonstrated potential value for identifying myocardial injury, plaque instability, ventricular remodeling, and residual cardiovascular risk [21]. Future iterations of the AIRA-CVD framework may incorporate these emerging diagnostic indices as clinical evidence continues to evolve [21].

Recent evidence demonstrates that AI consistently improves cardiovascular prediction compared with many conventional statistical approaches by integrating complex multidimensional clinical information. Contemporary reviews have reported that numerous validated machine-learning algorithms achieve discrimination exceeding an AUC-ROC of 0.80 while improving diagnostic yield by approximately 15-20% in selected cardiovascular applications [22]. This paradigm shift highlights how multi-parametric computational architectures successfully decode complex translational data that traditional linear instruments underrepresent [22]. Nevertheless, model performance remains dependent upon population characteristics, data quality, external validation, and responsible clinical implementation.

A major strength of the proposed framework is its incorporation of inflammatory biomarkers into cardiovascular risk prediction. Traditional cardiovascular risk calculators have contributed substantially to preventive cardiology; however, they rely predominantly on demographic and clinical variables and may not fully capture the biological processes underlying disease progression [4]. Chronic vascular inflammation plays a central role in endothelial dysfunction, atherosclerosis, plaque progression, and plaque instability [5,15]. Biomarkers such as hs-CRP and IL-6, therefore, provide biologically relevant information that may complement conventional cardiovascular risk factors and improve identification of individuals at elevated risk before overt clinical manifestations occur [6,8].

The framework also leverages advances in AI and machine learning to integrate complex healthcare data sources [9,10]. Recent developments in AI have demonstrated the potential of predictive analytics to improve disease detection, risk stratification, and clinical decision support across multiple medical specialties [11]. By combining longitudinal clinical information with inflammatory biomarker data, the proposed framework seeks to generate individualized cardiovascular risk assessments that may enhance preventive care and support earlier intervention strategies.

An additional distinguishing feature of the validation pathway is the incorporation of histopathological vascular remodeling as a biological validation layer. Many predictive algorithms are evaluated primarily using clinical outcomes without direct assessment of underlying disease mechanisms. By correlating biomarker signatures and machine learning predictions with tissue-level vascular pathology, the framework seeks to strengthen biological plausibility and improve confidence in model outputs [15,16]. This approach may help bridge the gap between computational prediction and measurable cardiovascular pathology.

Responsible implementation of AI in healthcare requires careful consideration of transparency, fairness, and clinical oversight. Previous work examining ethical governance of cardiovascular AI highlighted the importance of explainability, accountability, and bias mitigation in healthcare applications [13]. Accordingly, the AIRA-CVD validation framework incorporates explainable AI methodologies and emphasizes human-in-the-loop decision-making to support clinician trust and appropriate interpretation of predictive outputs [18]. Continuous monitoring of model performance across diverse populations is also proposed to promote equitable implementation and reduce the risk of algorithmic bias [19].

The framework may have broader implications for public health and CVD prevention. CVD continues to impose substantial clinical and economic burdens, while modifiable risk factors such as hypertension, obesity, physical inactivity, and poor dietary habits remain highly prevalent [1,3]. Recent work examining lifestyle determinants of hypertension further underscores the importance of integrating biological and population-health perspectives when addressing cardiovascular risk [14]. If successfully validated, the AIRA-CVD framework could support both precision medicine initiatives and broader cardiovascular prevention strategies by facilitating earlier identification of high-risk individuals and improving allocation of preventive resources [20].

The COVID-19 pandemic substantially disrupted routine cardiovascular screening, outpatient follow-up, and preventive care, contributing to delayed diagnosis and management of coronary artery disease and heart failure. Furthermore, persistent cardiovascular complications associated with long COVID continue to affect patients worldwide, increasing the demand for scalable approaches capable of identifying individuals at elevated risk despite healthcare resource limitations. Within this context, the reach of AI-enabled frameworks and digital health tools holds immense potential to bridge care gaps. By optimizing risk stratification pipelines remotely and processing heterogeneous medical registries automatically, such frameworks can enhance population-level surveillance, facilitate earlier identification of high-risk individuals, and assist clinicians in prioritizing preventive interventions during periods of constrained healthcare access.

Several limitations should be acknowledged. First, the proposed framework remains conceptual and has not yet undergone prospective clinical validation. Consequently, its predictive performance, clinical utility, and impact on patient outcomes remain to be established through future studies. Second, successful implementation will depend on the availability of high-quality, interoperable clinical, biomarker, imaging, and pathology datasets. Third, variability in biomarker measurement, healthcare infrastructure, and patient population characteristics may influence model generalizability across different clinical settings. Additionally, challenges related to data privacy, regulatory oversight, algorithmic bias, and integration within existing clinical workflows must be addressed before widespread adoption can occur [17-19]. Despite these limitations, the proposed validation pathway provides a structured roadmap for future investigation and implementation of biologically informed AI systems in cardiovascular medicine.

Unlike conventional cardiovascular risk calculators that rely primarily on demographic and clinical variables or standalone machine-learning algorithms, the AIRA-CVD framework uniquely combines inflammatory biomarkers, biological verification through opportunistic histopathology, explainable AI, algorithmic fairness assessment, and human-in-the-loop clinical oversight within a single translational validation pathway. This integrated architecture is intended not as a replacement for existing risk prediction models but as a structured framework for future biologically informed precision cardiovascular medicine.

## Conclusions

This technical report proposes a structured clinical validation pathway for the AIRA-CVD rather than reporting completed clinical validation. Building upon prior work, the framework proposes a four-phase pathway encompassing retrospective model development, inflammatory biomarker integration, histopathological validation, and prospective clinical implementation. By integrating clinical data, inflammatory biomarkers, machine learning analytics, and biological validation strategies, the framework seeks to advance cardiovascular risk assessment beyond conventional prediction models. The incorporation of explainable AI and human-in-the-loop oversight further supports responsible implementation within clinical practice.

Although prospective validation remains necessary, the proposed framework provides a structured roadmap for future development of biologically informed AI systems in cardiovascular medicine. If successfully validated, AIRA-CVD may contribute to earlier detection of cardiovascular risk, improved preventive interventions, and advancement of precision cardiovascular care.

## References

[1] (2026). Centers for Disease Control and Prevention. Heart disease facts. https://www.cdc.gov/heart-disease/data-research/facts-stats/index.html.

[2] (2026). Centers for Disease Control and Prevention. Fast facts: health and economic costs of chronic conditions. https://www.cdc.gov/chronic-disease/data-research/facts-stats/index.html.

[3] Martin SS, Aday AW, Allen NB (2025). 2025 heart disease and stroke statistics: a report of US and global data from the American Heart Association. Circulation.

[4] Arnett DK, Blumenthal RS, Albert MA (2019). 2019 ACC/AHA guideline on the primary prevention of cardiovascular disease: a report of the American College of Cardiology/American Heart Association Task Force on Clinical Practice Guidelines. Circulation.

[5] Libby P (2002). Inflammation in atherosclerosis. Nature.

[6] Ridker PM (2007). C-reactive protein and the prediction of cardiovascular events among those at intermediate risk: moving an inflammatory hypothesis toward consensus. J Am Coll Cardiol.

[7] Ridker PM, Everett BM, Thuren T (2017). Antiinflammatory therapy with canakinumab for atherosclerotic disease. N Engl J Med.

[8] Ridker PM, Danielson E, Fonseca FA (2008). Rosuvastatin to prevent vascular events in men and women with elevated C-reactive protein. N Engl J Med.

[9] Rajkomar A, Dean J, Kohane I (2019). Machine learning in medicine. N Engl J Med.

[10] Topol EJ (2019). High-performance medicine: the convergence of human and artificial intelligence. Nat Med.

[11] Johnson KW, Torres Soto J, Glicksberg BS (2018). Artificial intelligence in cardiology. J Am Coll Cardiol.

[12] Ogbuefi C (2026). Artificial Intelligence-Driven Integrated Risk Assessment of Cardiovascular Disease (AIRA-CVD): a technical framework incorporating inflammatory biomarker signatures and histopathological vascular remodeling. Cureus.

[13] Ogbuefi C, Ezika OL, Egbunike JO, Ogbuefi KE (2026). Ethical governance of artificial intelligence in cardiovascular disease management: a health policy perspective. Cureus.

[14] Ogbuefi C, Ezika O, Ogbuefi K, Frank-Okeke NN (2026). Modifiable lifestyle determinants of hypertension and cardiovascular risk in southeastern Nigeria: a narrative review from a socioecological perspective. Cureus.

[15] Ross R (1999). Atherosclerosis — an inflammatory disease. N Engl J Med.

[16] Virmani R, Burke AP, Farb A, Kolodgie FD (2006). Pathology of the vulnerable plaque. J Am Coll Cardiol.

[17] Obermeyer Z, Powers B, Vogeli C, Mullainathan S (2019). Dissecting racial bias in an algorithm used to manage the health of populations. Science.

[18] Char DS, Shah NH, Magnus D (2018). Implementing machine learning in health care — addressing ethical challenges. N Engl J Med.

[19] (2026). WHO. Ethics and governance of artificial intelligence for health. https://www.who.int/publications/i/item/9789240029200.

[20] (2026). Million Hearts. About Million Hearts® 2027. https://millionhearts.hhs.gov/about-million-hearts/index.html.

[21] Mittal A, Singhal K, Djoumsie EBG, Sharma YP, Batta A (2026). Emerging novel biomarkers in acute coronary syndrome management: insights and future directions. Cardiovasc Innov Appl.

[22] Parizad R, Hatwal J, Brar A, Desai R, Batta A, Mohan B (2026). Artificial intelligence for cardiovascular risk prediction: an umbrella review of applications and translational challenges. Vasc Health Risk Manag.

